# C-Peptide as a Therapy for Type 1 Diabetes Mellitus

**DOI:** 10.3390/biomedicines9030270

**Published:** 2021-03-08

**Authors:** Rachel L. Washburn, Karl Mueller, Gurvinder Kaur, Tanir Moreno, Naima Moustaid-Moussa, Latha Ramalingam, Jannette M. Dufour

**Affiliations:** 1 Department of Immunology and Infectious Disease, Texas Tech University Health Sciences Center, Lubbock, TX 79430, USA; rachel.washburn@ttuhsc.edu; 2 Department of Cell Biology and Biochemistry, Texas Tech University Health Sciences Center, Lubbock, TX 79430, USA; karl.mueller@ttuhsc.edu (K.M.); tanir.moreno@ttuhsc.edu (T.M.); 3 Departments of Medical Education, Cell Biology and Biochemistry, Obesity Research Institute, Texas Tech University Health Sciences Center, Lubbock, TX 79430, USA; gurvinder.kaur@ttuhsc.edu; 4 Department of Nutritional Sciences, College of Human Sciences, Obesity Research Institute, Texas Tech University, Lubbock, TX 79409, USA; naima.moustaid-moussa@ttu.edu; 5 Department of Nutrition and Food Studies, Falk College, Syracuse University, Syracuse, NY 13244, USA; lramalin@syr.edu; 6 Departments of Immunology and Molecular Microbiology, Texas Tech University Health Sciences Center, Lubbock, TX 79430, USA; 7 Departments of Medical Education, Obesity Research Institute, Texas Tech University Health Sciences Center, Lubbock, TX 79430, USA

**Keywords:** diabetes mellitus, C-peptide, endothelial dysfunction, endothelial activation, cardiovascular disease, Sertoli cells

## Abstract

Diabetes mellitus (DM) is a complex metabolic disease affecting one-third of the United States population. It is characterized by hyperglycemia, where the hormone insulin is either not produced sufficiently or where there is a resistance to insulin. Patients with Type 1 DM (T1DM), in which the insulin-producing beta cells are destroyed by autoimmune mechanisms, have a significantly increased risk of developing life-threatening cardiovascular complications, even when exogenous insulin is administered. In fact, due to various factors such as limited blood glucose measurements and timing of insulin administration, only 37% of T1DM adults achieve normoglycemia. Furthermore, T1DM patients do not produce C-peptide, a cleavage product from insulin processing. C-peptide has potential therapeutic effects in vitro and in vivo on many complications of T1DM, such as peripheral neuropathy, atherosclerosis, and inflammation. Thus, delivery of C-peptide in conjunction with insulin through a pump, pancreatic islet transplantation, or genetically engineered Sertoli cells (an immune privileged cell type) may ameliorate many of the cardiovascular and vascular complications afflicting T1DM patients.

## 1. Diabetes Mellitus

Diabetes mellitus (DM) is a chronic metabolic disorder caused by insufficient production of insulin or insulin resistance, which results in elevated blood glucose levels. The worldwide increase in DM is alarming, with rates up from 108 million in 1980 to 422 million in 2014 [1,2]. In the United States (US) alone, 34.2 million people (10.5% of the population) have DM and an additional 88 million have prediabetes; therefore, one third of the US population has some form of DM [1,3]. In 2016, 1.6 million deaths worldwide were directly attributed to DM, and roughly 80,000 people died of DM in the US in 2015 [4,5]. Hence, DM accounts for approximately 3% of all deaths in the US, making it the seventh leading cause of death [6]. However, this is a low estimate, with DM actually making up closer to 12% of all deaths, since diabetes-related cardiovascular disease (CVD), renal disease, or hyperglycemia are far underreported on death certificates [3,7].

DM is classically divided into two main subsets: Type 1 diabetes mellitus (T1DM) and Type 2 diabetes mellitus (T2DM), with T2DM being the most prevalent, afflicting 90–95% of patients with DM [8]. Both T1DM and T2DM are characterized by chronic hyperglycemia, which initiates the pathologies and comorbidities associated with DM such as CVD, renal failure, amputations, and retinopathy [8,9,10].

T1DM is an autoimmune disease characterized by self-reactive immune cell destruction of the insulin-producing beta cells within the pancreatic islets. Insulin is an important regulator of glucose homeostasis, and the loss of this vital hormone results in hyperglycemia and the pathologies associated with T1DM. T1DM affects about 1.25 million Americans, including approximately 200,000 children [11]. Roughly 30,000 people are diagnosed with T1DM each year, and the incidence among children is increasing at a rate of 3% each year [12,13]. The financial burden of T1DM alone in the US is estimated at $15 billion dollars annually [12]. The goal of DM therapy is to normalize blood glucose levels and reduce associated chronic co-morbidities. As patients with T1DM no longer produce insulin, they require lifelong insulin replacement therapies by either multiple insulin injections or continuous insulin administration via an insulin pump [14,15]. Without regular administration of insulin, these patients will develop diabetic ketoacidosis (DKA) and can die from hyperglycemic complications. Unfortunately, even when insulin is administered as directed, hyperglycemia is very difficult to control [16]. Several issues exist with insulin-only replacement therapies that include inadequate timing and/or dosage of insulin administration, limitations of blood glucose testing, hepatic processing of peripherally administered insulin disparities, and rising cost of insulin therapies [17]. These treatment obstacles lead to only 37% of adults with T1DM achieving normalized blood glucose levels as well as healthy ranges of cholesterol and blood pressure [18].

Unlike T1DM being characterized by loss of insulin-producing cells, T2DM is typified by insulin resistance where cells, especially muscle and adipose tissue, no longer respond to insulin appropriately, which lowers the glucose clearance from the blood [9]. Initially, patients with T2DM develop insulin resistance and are hyperinsulinemic. Overtime, excess burden on pancreatic beta cells to continuously secrete more insulin to compensate for resistance leads to beta cell dysfunction and eventually beta cell death and decrease in insulin production [19,20]. By analyzing pancreatic tissue sections taken during autopsies, it is estimated that there is approximately a 40 to 60% decrease in beta cell mass in T2DM patients compared to nondiabetic controls [21]. Even with this decrease, the remaining beta cells still produce insulin. Hence, DKA is less common among patients with T2DM [22]. Risk factors for developing T2DM include genetics, ethnicity, environment, obesity, physical inactivity, prediabetes, and gestational diabetes. In patients diagnosed with prediabetes or T2DM, lifestyle modifications including a healthy diet and exercise accompanied by hypoglycemic agents, like metformin, are often prescribed as first-line treatment options [23,24]. If caught early enough, lifestyle changes along with hypoglycemic agents may be enough to stop and even reverse disease progression. However, as the disease progresses, most T2DM patients require further intervention, including insulin replacement therapy [25]. Unfortunately, prevalence of T2DM is increasing and it is estimated that one third of individuals born in the year 2000 will develop diabetes during their lifetime; this increases to 50% if they are of a high risk minority [11].

## 2. Metabolic Dysregulation

**Figure 1:**
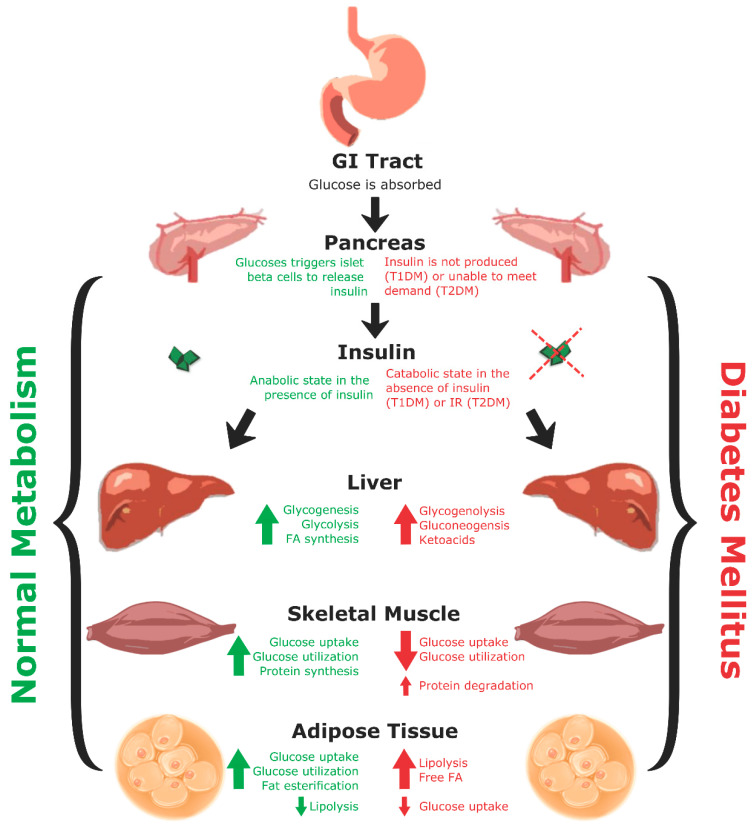
A comparison of normal metabolism to metabolism in DM. Under conditions of normal metabolism, glucose is absorbed from the GI tract and triggers pancreatic islet beta cells to release the hormone insulin. Insulin initiates a series of anabolic events in the liver, skeletal muscle, and adipose tissue. In the liver, insulin causes an increase of glucose utilization and FA synthesis. In skeletal muscle, it increases rates of protein synthesis, glucose utilization, and fuel uptake while decreasing rates of protein degradation. In adipose tissue, insulin increases rates of glucose uptake and utilization, fat esterification, and fuel uptake while decreasing lipolysis [16,28]. The opposite effects are seen under DM conditions, where lack of insulin or insulin resistance (IR) favors catabolic events. In DM, glycogenolysis and gluconeogenesis pathways are activated in the liver, ketoacid production increases in the liver (T1DM), glucose uptake is decreased in skeletal muscle and adipose tissue, protein degradation is increased in skeletal muscle, and lipolysis and free FA release are increased in adipose tissue [28].

**Figure 2:**
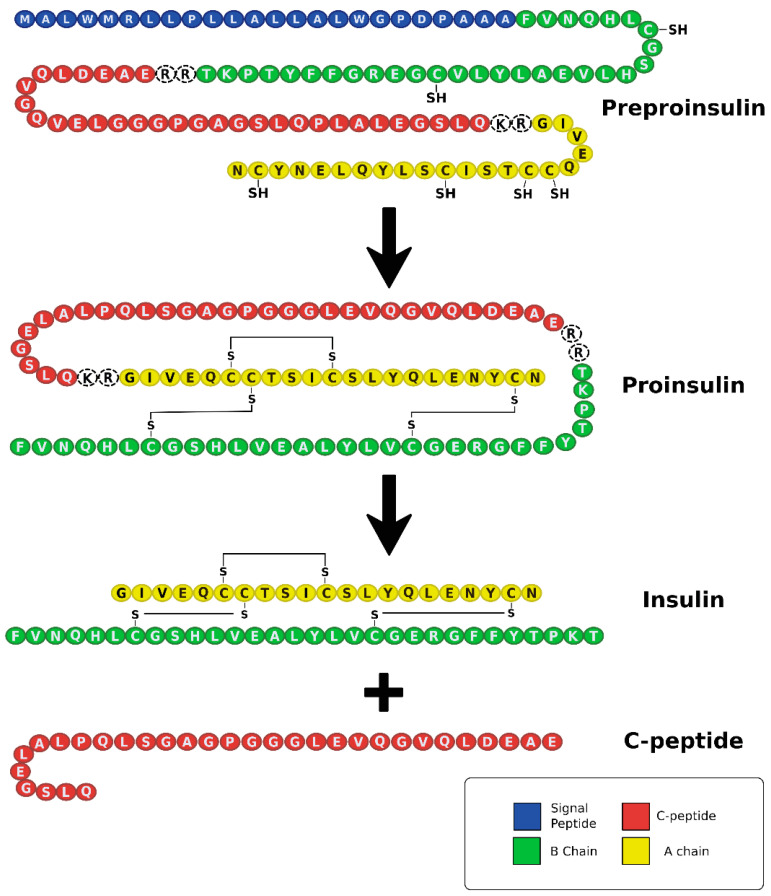
Post-translational modifications of human preproinsulin to produce insulin and C-peptide. After translation of preproinsulin, the signal sequence is cleaved from the main molecule in the rough endoplasmic reticulum, leaving proinsulin. Proinsulin then undergoes folding and three disulfide bonds are formed. Proinsulin is transported to the Golgi apparatus where it is packaged in secretory granules. Within the secretory granules, C-peptide is cleaved from the proinsulin molecule by prohormone convertases (PC) 1 and 2. This cleavage results in the removal of four amino acids (KR and RR) from the final products. PC1 and PC2 are almost exclusively found in pancreatic islet beta cells. C-peptide and the mature insulin molecule are secreted together from the secretory granules [26].

A recurring commonality between T1DM and T2DM is metabolic dysregulation causing elevated blood glucose levels (Figure 1). Under normal conditions, after eating a meal, glucose is absorbed through the gastrointestinal (GI) tract leading to an increase in blood glucose levels. This increase in blood glucose is sensed by beta cells located in pancreatic islets, which then secrete insulin stored within secretory granules. Insulin is produced from a precursor protein preproinsulin that consists of a signal peptide, A and B chains, and C-peptide (Figure 2) [26,27]. The signal sequence is cleaved from preproinsulin in the rough endoplasmic reticulum to produce proinsulin. Proinsulin is then transported through the Golgi apparatus to the secretory granules where it is further processed into insulin and C-peptide. Since insulin and C-peptide are produced from the same precursor protein, they are secreted into circulation in equimolar amounts. Patients with T1DM lack both insulin and C-peptide whereas patients with T2DM initially produce high amounts of insulin and C-peptide, and these levels vary as the disease progresses.

Insulin is a critical regulator of metabolism that maintains the body in an anabolic state (Figure 1) [28]. In the liver, glycogenesis, glycolysis, and fatty acid (FA) synthesis are increased, while gluconeogenesis and glycogenolysis are inhibited. In skeletal muscle, glucose uptake, glucose utilization, and protein synthesis are stimulated, whereas proteolysis is decreased. In adipose tissue, glucose uptake, glucose utilization, and fat synthesis are increased while lipolysis is decreased [16,28]. Conversely, in DM a lack of insulin (as in T1DM) or insulin resistance (as in T2DM) creates a hyperglycemic environment causing metabolism to switch from an anabolic to a catabolic state (Figure 1) [28]. The liver experiences increased glycogenolysis and gluconeogenesis, while skeletal muscle and adipose tissue experience decreased glucose uptake and utilization. Moreover, skeletal muscle experiences increased protein degradation, and adipose tissue has increased rates of lipolysis leading to elevated circulating levels of glucose, and free FA.

**Figure 3:**
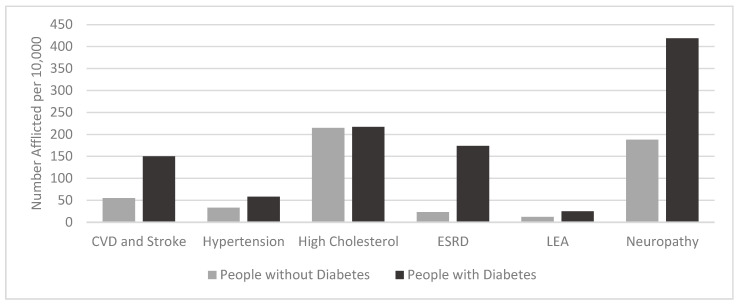
Common comorbidities and complications of DM in the US population as compared to the incidence in those not afflicted with DM [8,33,34,35,36,37,38,39]. At least 34.2 million Americans have DM. DM was directly attributed to about 80,000 deaths in the US. and it was a contributing cause for an additional 252,806 deaths in 2015 [8]. ESRD: End Stage Renal Disease. LEA: Lower Extremity Amputation.

In both T1DM and T2DM, hyperglycemia and metabolic dysregulation encourage oxidative stress and a pro-inflammatory environment [19]. This promotes endothelial damage, which disrupts vasculature and brings about varying complications and comorbidities associated with DM (Figure 3) [19]. Complications include: coronary artery disease (CAD), CVD, stroke, renal disease, peripheral neuropathy, diabetic retinopathy, lower extremity amputation, hypertension, dyslipidemia, hearing impairment, obstructive sleep apnea, dementia, and increased incidences of specific cancers [12,13,29,30]. These complications vary in frequency and severity between the two types of DM, with microvascular and macrovascular issues being more prevalent in T1DM [10,29,31,32]. This may be because patients with T1DM experience more severe metabolic dysregulation and also lack C-peptide, which has been found to decrease damage to the vasculature endothelium.

## 3. Endothelial Damage

**Figure 4:**
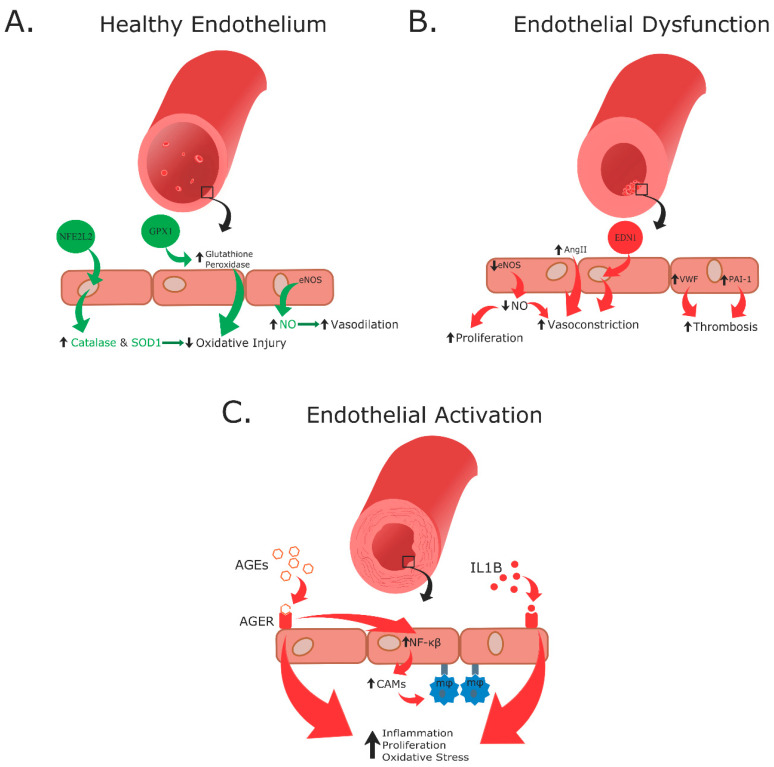
Effects of hyperglycemia leading to endothelial damage. Healthy endothelium (**A**) has signaling through the nuclear factor erythroid 2-related factor 2 (NFE2L2) pathway which increases production of catalase and superoxide dismutase (SOD1). Additionally, glutathione peroxidase 1 (GPX1) increases expression of glutathione peroxidase. Both of these processes decrease oxidative injury. Furthermore, endothelial nitric oxide synthase (eNOS) produces increased quantities of nitric oxide (NO), which in turn increases vasodilation allowing for healthy circulation. During endothelial dysfunction (**B**), hyperglycemia increases production of vasoactive substances including endothilin-1 (EDN1, a vasoconstrictor), von Willebrand factor (VWF, a circulating glycoprotein involved in coagulation), and plasminogen activator inhibitor-1 (PAI-1, an inhibitor of fibrinolysis). Thrombosis is increased during endothelial dysfunction through an increase in VWF and PAI-1. Endothelial cell proliferation and vasoconstriction are promoted by a decrease in NO production by eNOS [41,42,43]. Additionally, an increase in angiotensin II (Ang II) pathway activation further promotes vasoconstriction. Endothelial activation (**C**) occurs when advanced glycation end-products (AGEs) bind AGEs Receptor (AGER) and activate the nuclear factor-κB (NF-κB) signaling pathway [44,45,46,47]. This upregulates expression of inflammatory markers and cellular adhesion molecules like intracellular adhesion molecule-1 (ICAM-1) and vascular cell adhesion molecule-1 (VCAM-1) to encourage macrophage (MΦ) adhesion and chemotaxis [41,48,49,50]. Moreover, IL-1B binds to its receptor on the endothelium and, along with AGEs binding, causes inflammation, proliferation, and oxidative stress.

Vasculature, comprised of arteries, veins, and capillaries, carries blood throughout the body and is lined with a single layer of endothelial cells covered by smooth muscle cells. Normally, the endothelial layer along with the smooth muscle maintains vascular tone, blood vessel structure, and vascular homeostasis. The endothelium accomplishes this through regulation of various cellular functions including cell adhesion, inflammatory molecule modulation, immune system response, metabolism, smooth muscle cell proliferation, vascular permeability, and coagulation (Figure 4A) [20]. 

In patients with DM, vascular homeostasis becomes disrupted thus causing endothelial dysfunction, which favors a chronic vasoconstrictive environment and thrombosis (Figure 4B). Additionally, increase in adhesion and inflammatory factor production leads to endothelial immune activation (Figure 4C). Moreover, oxidative stress occurs, which subsequently causes an increase in reactive oxygen species (ROS) production. These processes culminate in endothelial cell detachment into circulation, increased levels of apoptotic cell death, and decreased endothelial survival. Together, these events lead to microvascular (small vessels) and macrovascular (large vessels) pathologies such as hypertension, CAD, CVD, and atherosclerosis [20,40,41].

### 3.1. Endothelial Dysfunction

Endothelial dysfunction occurs when hyperglycemia deceases production of vasodilators, particularly nitric oxide (NO), by decreasing activity of endothelial nitric oxide synthase (eNOS) (Figure 4B). eNOS is the primary enzyme responsible for synthesizing NO from L-arginine and requires several cofactors such as nicotinamide adenine dinucleotide phosphate (NADPH), coenzyme Q10, and tetrahydrobiopterin. If the levels of these cofactors diminish, eNOS becomes uncoupled and produces superoxide instead of NO, which can combine to produce peroxynitrite [51]. Endothelial-produced NO is critical for the maintenance of normal vascular tone [20]. 

**Figure 5:**
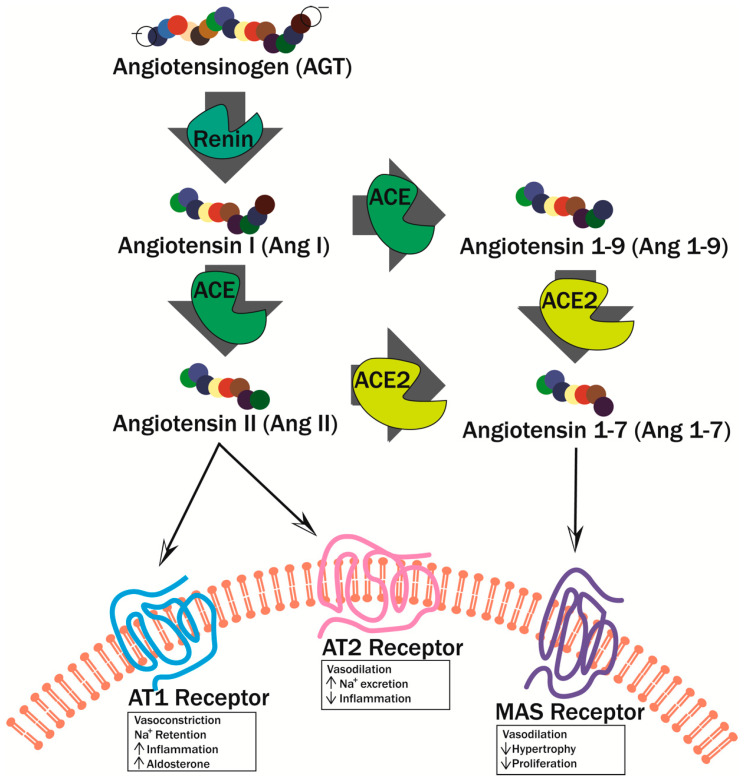
Renin–angiotensin pathway. Angiotensin is cleaved by renin to form Ang I. Ang I is cleaved by angiotensin-converting enzyme (ACE) into either Ang II or angiotensin 1–9 (Ang 1–9). Ang II can bind two different receptors: AT1 Receptor and AT2 Receptor. When Ang II binds AT1R, it initiates vasoconstriction, sodium retention, increased inflammation, and increased aldosterone. When Ang II binds AT2R, then vasodilation, sodium excretion, and decreased inflammation occur. Ang II and Ang 1–9 are also converted to Ang 1–7 by ACE2. Ang 1–7 binds the MAS1 oncogene (MAS) receptor where it triggers vasodilation and decreases hypertrophy and proliferation.

While the production of vasodilators decreases during hyperglycemia, vasoconstrictor production is increased, specifically endothilin-1 (EDN1) and angiotensin II (Ang II). Not only does EDN1 directly cause vasoconstriction, but it also decreases NO production and further exasperates endothelial dysfunction [52]. Additionally, EDN1 is involved in other pathophysiological processes associated with long term DM such as pulmonary fibrosis, hypertension, and atherosclerosis. Ang II, when bound to the Ang II Type 1 (AT1) Receptor, elicits a proinflammatory effect through vasoconstriction and sodium retention (Figure 5) [52]. Conversely, when Ang II binds to the Ang II Type 2 (AT2) Receptor, an anti-inflammatory effect is elicited through vasodilation and sodium secretion (Figure 5) [52].

Furthermore, endothelial dysfunction causes overproduction of coagulation-promoting factors like von Willebrand factor (VWF) and plasminogen activator inhibitor-1 (PAI-1), which is also further induced by Ang II [53,54]. Elevated levels of VWF tend to precede microalbuminuria and lead to increased permeability of blood capillaries, as well as inflammation. Elevated levels of PAI-1 lead to decreased fibrinolysis and increased fibrosis [55]. Taken together, increased production of these vasoactive substances leads to chronic blood flow abnormalities, and thrombosis [20,40]. 

### 3.2. Endothelial Activation

Endothelial activation is the shift of endothelial cells towards a proinflammatory state. As vasculature becomes more permeable and immune cell infiltration increases, a low-grade inflammatory environment develops that promotes endothelial activation (Figure 4C) [56]. Release of factors from the endothelium, such as vascular endothelial growth factor (VEGF), contributes to increasing vascular permeability, which allows immune cells to infiltrate the area. In addition, cellular adhesion molecules (CAMs) including vascular cellular adhesion molecule-1 (VCAM-1), intracellular adhesion molecule-1 (ICAM-1), P-selectin (CD62P), and E-selectin (CD62E), when expressed on endothelial tissue surfaces, incite immune cell infiltration by allowing for trafficking and extravasation of leukocytes into the subendothelial space [20,40,57,58]. Moreover, endothelial cells that upregulate CAMs promote monocyte adherence and differentiation into macrophages. The macrophages then phagocytose oxidized low-density lipoproteins (LDL) released in the arterial wall and become foam cells. As foam cells accumulate in the artery wall, this promotes plaque formation which is an early event in atherosclerosis development [59].

Hyperglycemia also leads to endothelial activation directly. Excess glucose availability due to chronic hyperglycemia, leads to production of advanced glycation end products (AGEs). AGEs are linked to elevated inflammation, oxidative stress, smooth muscle cell proliferation, and endothelial cell apoptosis [47]. Hyperglycemia results in inflammation since AGEs bind to receptor for AGEs (AGER) and activate the NF-κB pathway to upregulate production of both CAMs and proinflammatory cytokines [48,60]. The activated endothelial phenotype of low-grade inflammation due to AGEs activation of the NF-κB pathway is often seen in T1DM and is characterized by expression of CAMs [20,40,57]. 

### 3.3. Oxidative Stress

Oxidative stress is the molecular upstream event that triggers diabetic complications in endothelial cells. Hyperglycemia leads to oxidative stress through various mechanisms including overproduction of AGEs (as described above), eNOS uncoupling, inflammatory pathways, excess glucose flooding into accessory pathways (such as the polyol and hexosamine pathways), and through the electron transport chain of aerobic metabolism. 

Interestingly, besides vasodilation, NO also inhibits inflammation and cell adhesion, smooth muscle cell proliferation, apoptosis, and thrombosis [20]. Creation of peroxynitrite decreases available NO levels, thus encouraging a proinflammatory, vascular vasoconstrictive environment conducive to atherosclerosis, thrombosis, and atherogenesis [20,40]. This is supported by the fact that endothelial cells in atherosclerosis have characteristically low NO bioavailability and elevated ROS levels, further contributing to endothelial dysfunction and activation as previously described [40,49].

Increased glucose leads to a flux in polyol and hexosamine biosynthetic pathways producing detrimental levels of ROS molecules [20,61,62]. Furthermore, hyperglycemia can directly trigger a shift toward increased oxidative stress by decreasing antioxidant production and thereby increasing ROS. This is due to an increase in levels of NADPH through polyol and metabolic pathways. NADPH is a reduced molecule that plays a role in aerobic metabolism and immune cell free radical production of respiratory burst, which functions to eliminate pathogens [63]. NADPH oxidase or eNOS uncoupling catalyze the transfer of electrons from NADPH to oxygen, forming superoxide [63]. Left unchecked, ROS generated by NADPH in this pathway can further exasperate vascular endothelial oxidative stress [20]. NADPH oxidase, found in membranes of endothelial cells, is the largest source of superoxide production and is activated by protein kinase C beta (PKCβ), EDN1, Ang II, and growth factors such as TGF-β [20,40,46]. Under normal physiological conditions, PKCβ is activated by the lipolysis product diacylglycerol (DAG). Under conditions associated with DM, elevated levels of free FAs circulate within blood along with excess glucose. This leads to increased levels of DAG production and increased activation of PKCβ, which goes on to increase the production of ROS by NADPH oxidase [40,46,64]. 

Furthermore, oxidative stress leads to decreased activity of the sodium potassium ATPase, an intermembrane pump critically important for nerve signaling, nerve conduction, and kidney function [65,66]. With an increase in ROS levels, subunits of the sodium potassium ATPase become oxidized, which not only inhibits its activity, but also leads to the degradation of the pump itself [67]. Decrease in activity of sodium potassium ATPase plays a critical role in the development and maintenance of diabetic neuropathy pathogenesis [68]. Ultimately, excessive ROS levels can alter normal cellular signaling within the endothelium vasculature eventually leading to cell death. 

### 3.4. Cell Death

Overall, decreased NO, inflammation, AGEs production, and oxidative stress work synergistically with hyperglycemia to create an environment that favors endothelial cell apoptosis and vascular pathology. ROS target DNA, lipids, and proteins, specifically mitochondrial DNA, causing cellular damage [69]. As disease progresses, an imbalance in cell repair versus cell death damages the microvasculature, which can lead to the development of hypoxic areas within the tissue [50]. Microvascular disease including accelerated atherogenesis, myocardial infarction, stroke, and peripheral vascular disease are often inevitably experienced by T1DM patients [6,32].

When left unchecked, hypoxia-induced neovascularization initiates macrovascular damage [69]. In the case of diabetes, these mechanisms contribute to renal failure, retinopathy, CVD, and atherosclerosis (the restriction of blood flow due to excess deposition of cholesterol and fats on arterial surfaces) [70]. Macrovascular disease is a critical complication of DM, with diabetic CVD as the leading cause of death in this patient population [10,13,20,71]. Glycemic control through insulin replacement therapies can reduce diabetes-associated vascular complications, but even intensive glycemic control does not normalize the risk of developing these comorbidities [29,72,73]. In addition to lacking insulin, T1DM patients lack C-peptide, a molecule produced from posttranslational modification of insulin, which is normally present in the healthy population and among patients with T2DM [41]. 

## 4. C-Peptide

C-peptide is a 31-amino acid linker chain cleaved from proinsulin by prohormone convertase enzymes in beta cells to produce the mature, functional insulin hormone (Figure 2) [74]. Since C-peptide is secreted in equimolar concentrations with insulin and has almost a five-times longer half-life than insulin, it is used as a clinical marker for pancreatic beta cell function [75,76,77]. Though traditionally considered a simple, biologically inert molecule, current research reveals that C-peptide is a biologically active hormone that has therapeutic potential for treatment of complications associated with diabetes [77]. 

### 4.1. C-Peptide in T1DM

Small and large vessels course throughout the body, as such, micro- and macro-vascular complications associated with T1DM pathogenesis affect the majority of tissues and organs within the body, especially the kidneys, peripheral nerves, blood vessel endothelium, and capillaries of peripheral limbs. As discussed previously, many of these complications arise from oxidative stress, inflammation, thrombosis, and cell destruction initiated by endothelial dysfunction and activation.

**Figure 6:**
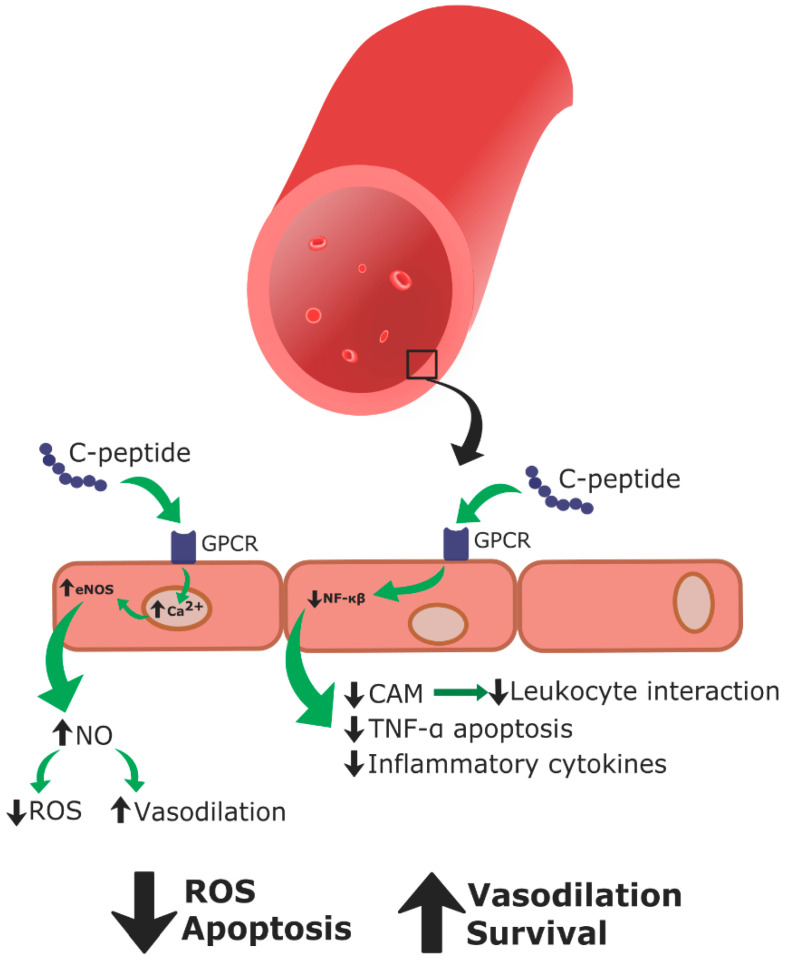
C-peptide action on endothelial cells. C-peptide binds to its G-protein coupled receptor (GPCR) to increase intracellular calcium levels. This leads to an increase in eNOS production of NO to decrease ROS and increase vasodilation. Furthermore, C-peptide decreases NF-κB activity to decrease CAM expression and leukocyte interaction, TNF-α mediated apoptosis, and production of inflammatory cytokines. Taken together, C-peptide decreases ROS production and apoptosis of endothelial cells while increasing vasodilation and endothelial cell survival.

C-peptide replacement therapy as a treatment for T1DM vascular pathologies has been studied in vitro, ex vivo, and in vivo (in T1DM rodent models, as well as some clinical human studies involving T1DM patients (summarized in Table 1)). C-peptide therapy in streptozotocin-induced diabetic rats led to general vascular improvements, along with improved renal and neural vasculature function [78]. Improvements in vascular impairment occurred by preventing disruption of the sodium potassium ATPase, which also prevented progressed states of endothelial dysfunction [78,79]. In vitro, C-peptide increased intracellular concentrations of calcium, which in turn increased eNOS activity and synthesis of NO. This further prevented endothelial dysfunction (Figure 6) [80]. Along these lines, C-peptide encourages vasodilation through increased NO production [81].

These results were also observed in T1DM patients, as blood flow to the extremities and skin were improved when insulin was co-administered with C-peptide [82]. During episodes of endothelial dysfunction, C-peptide was found to decrease endothelial surface expression of CAMs, specifically P-selectin and ICAM-1, and inhibit leukocyte interaction with the endothelium, which improved complications associated with endothelial activation (Figure 6) [83]. In this instance, C-peptide operated through a NO-dependent mechanism to downregulate endothelial CAM expression ([82,83], reviewed in [44]).

Regarding isolated human erythrocytes, C-peptide replacement therapy increased sodium potassium ATPase activity in red blood cells of human patients with T1DM, improving erythrocyte deformability [84,85,86]. C-peptide has been shown to decrease ROS generation, protecting the endothelium against excess blood glucose and TNF-α induced apoptosis (Figure 6) [45]. Additionally, C-peptide has been shown to significantly reduce hyperglycemia-induced production of inflammatory chemokines and CAMs in endothelial cells (Figure 6) [87,88].

In rat renal tubular cells, C-peptide replacement increased sodium potassium ATPase activity, both in vitro and in vivo [43]. When C-peptide was administered to diabetic rats for 140 min, glomerular hyperfiltration and renal function were improved [89]. Further studies have shown that C-peptide administered to streptozotocin-diabetic rats for two to four weeks ameliorated glomerular hyperfiltration and albuminuria by reducing glomerular size [90,91]. Sodium potassium ATPase and eNOS activity is increased in the presence of C-peptide, and these two enzymes are characteristically decreased in patients with DM. Furthermore, C-peptide increases intracellular calcium ion concentrations, and in vitro models have shown that C-peptide increases the presence of eNOS and NO in endothelial cells [102,103].

In humans, diabetes-related nephropathy, including glomerular hyperfiltration, glomerular hypertrophy, and albuminuria have been improved by C-peptide administration in T1DM cases [104]. Short-term intravenous infusion of C-peptide overnight was shown to regulate renal function by reducing overall glomerular filtration rate and glucose utilization in these T1DM patients was even improved by overnight C-peptide infusions [94]. When C-peptide was administered in conjunction with insulin therapy to T1DM patients over a six month period in a double-blind placebo-controlled study, renal function was significantly improved [93,104]. Similarly, others have found that C-peptide reduces glomerular hyperfiltration and microalbuminuria, exerting beneficial effects on renal function [45].

With respect to the nervous system, after two weeks of therapy, C-peptide has been shown to increase the activity of sodium potassium ATPase in rat sciatic nerves [94]. C-peptide therapy stimulated nerve blood flow in streptozotocin-induced diabetic rats [94,95]. Meanwhile, in T1DM rats, two to eight months of C-peptide intervention partially prevented diabetic peripheral neuropathy and increased nerve fiber regeneration by fourfold [96].

In patients with T1DM, short-term administration of C-peptide by intravenous infusion for three hours improved overall autonomic nerve function [97]. Other studies found that when C-peptide was administered for three to six months in T1DM patients, peripheral neuropathy symptoms were improved as compared to placebo [72,93,98]. Moreover, sensory nerve conduction velocity as well as autonomic nerve function was increased in patients with T1DM when treated with C-peptide [93]. C-peptide also corrects endoneural blood flow through vasodilation, reducing hyperglycemia-induced neuronal changes [45].

The complete absence of C-peptide may effectuate increased cardiovascular risk in T1DM patients. Mechanistically, C-peptide may attenuate the inflammatory responses of insulin [105]. Canonically, insulin is thought of as an anti-inflammatory molecule, since insulin reduces hyperglycemia and thus decreases the oxidative stress and inflammation [44]. However, this is one side of the equation of insulin’s actions. In totality, insulin is much more than a simple glucoregulatory hormone. It is a major anabolic hormone that is associated with, and in fact necessitates, inflammatory responses [42,106]. In the context of a patient with T1DM, this effect may be negligible compared with the anti-inflammatory effect of decreasing hyperglycemia [106]. Though, this may explain a critical oversight as to why patients with T1DM still have an exaggerated cardiovascular risk even with insulin-mediated high glucose control.

Taken together, the results of the aforementioned studies lead to the conclusion that C-peptide is not simply an inert structural molecule, but instead elicits physiological effects, making it a biologically active hormone (Table 1, Figure 6). These evidences support the hypothesis that C-peptide together with insulin could improve endothelial activation and dysfunction in high glucose environments [45,77].

### 4.2. C-Peptide in T2DM

The beneficial effects of exogenously delivered C-peptide seem to be inconsequential in the physiology of healthy individuals, while the benefits for patients with T2DM are more complicated [44]. This may be explained by receptor saturation. The normal physiological concentration of C-peptide is between 0.5 and 2.0 ng/mL (0.3 and 0.6 nM) when fasting. Given the receptor saturation level of C-peptide is 1 nM, delivery of excess C-peptide would provide no additional benefit to individuals without DM. In T2DM patients, levels of C-peptide vary depending on the stage of the disease. Initially they have elevated levels, but levels then decrease as the disease progresses and the beta cells become dysfunctional and undergo apoptosis resulting in insulin and C-peptide deficiency. This could explain why C-peptide supplementation therapy has no effect on most non-T1DM populations, although delivery of C-peptide to T2DM patients with C-peptide deficiency might be beneficial [84].

Interestingly, the effects of C-peptide at elevated levels are controversial ([99,100,101], reviewed in [107]. In ex vivo studies using T cells from healthy individuals and thoracic artery tissue from T2DM patients, C-peptide levels of 10 nM exhibited proinflammatory behavior by increasing CD4 T cell chemotaxis to wound sites [99,100]. This increase of CD4 T cell chemotaxis has been observed in lesions associated with diabetic atherosclerosis at C-peptide concentrations over 1 nM [99,100]. Additionally, at concentrations of over 1 nM in 3T3 mouse fibroblast cells, C-peptide has been shown to actually increase activation of the NF-κB pathway, thus increasing transcription of proinflammatory genes [101]. As these studies were conducted using limited tissues (CD4 T cells, thoracic arterial tissue, and a mouse fibroblast cell line), these results could possibly be tissue specific. Overall, the effects of elevated C-peptide levels are still unclear and require further study, especially in T2DM patients.

### 4.3. Mechanism of Action

C-peptide’s mechanism of action is still contentious with a few probable hypotheses. Older literature posits that C-peptide could bind to the cell surface and create membrane pores, yet this suggestion was dismissed as C-peptide has five acidic amino acids. Thus, at physiologic pH, C-peptide has a negative five charge. Charged compounds typically do not associate with the phospholipid bilayer of the cell surface, and therefore the association of C-peptide with the cell surface is more likely with the peptide-binding domain of a membrane bound protein. This would refute the cell pore theory. In addition, there has been no evidence to support the creation of a membrane pore in vitro or in vivo [43].

There has been significant data to support the hypothesis that C-peptide’s mechanism of action is through a GPCR [45]. C-peptide’s effects are reversed by treatment with pertussis toxin, a GPCR inhibitor produced by the bacteria *Bordetella pertussis* [77]. Additionally, C-peptide works through GPCR second messengers like calcium, DAG, inositol triphosphate, and protein kinase C [43,108]. Given these data, it is more likely that C-peptide interacts with a GPCR to exert its effects. However, C-peptide is a small protein at 3.02 kDa, making crosslinking and isolation of a C-peptide receptor complex extremely difficult.

Recently, Kolar et al. made a breakthrough in C-peptide GPCR signaling [109]. Using bioinformatics, potential orphan GPCRs were narrowed down, leaving only GPCRs with binding sites likely to interact with C-peptide. Cells cloned with these orphan receptors were treated with C-peptide and second messengers were monitored, demonstrating that GPCR 146 was activated. These experiments lend credence to the hypothesis that C-peptide works through a GPCR mechanisms to deliver its effects [108,109].

Another possible mechanism by which C-peptide could employ its effects is by transition metal ion chelation. As mentioned previously, C-peptide is a highly negatively charged molecule [43]. Given its size and its negative nature, it is possible that C-peptide binds multivalent positive ions like transition metals such as iron, copper, or zinc [43,81,88,103,110]. This may exhibit a positive effect during hyperglycemia when large amounts of ROS are generated. The presence of a transition metal can be used as a catalyst for synthesis of ROS species from hydrogen peroxide. The binding of C-peptide to metal ions could lead to a critical reduction in ROS by removing metal ion catalysts and slowing the negative effects of hyperglycemia on endothelial cells to reduce end organ damage. While it is clear that C-peptide has beneficial effects, the ambiguity of its mechanism of action is a limiting factor that should be addressed in further research.

As we understand at this time, C-peptide binds in a stereospecific manner to a GPCR, which becomes saturated at 1 nM concentrations, with normal physiological concentrations between 0.3 to 0.6 nM during overnight fasting and between 1 to 3 nM after eating a meal [45,111]. However, in patients with T1DM, C-peptide concentrations are minimal to non-existent due to the loss of beta cells and insulin production. The lack of C-peptide in T1DM may be a major contributor to the development of microvascular and macrovascular complications in this patient population.

## 5. Physiological Delivery of C-Peptide

With these data in mind, T1DM can be considered a dual hormone deficiency disease that may be better treated by insulin and C-peptide combined replacement therapy, which would more closely approximate normal physiology [44,77,95,112,113]. As previously discussed, C-peptide has been shown to decrease generation of excess ROS, inhibit NF-κB activation, lower CAM and proinflammatory cytokine production, increase levels of NO, and activate sodium potassium ATPase, all of which result in decreased vascular dysfunction and improvements in the pathologies associated with T1DM. These observed functions demonstrate the potential of utilizing C-peptide as a therapy to treat the chronic complications of T1DM.

### 5.1. Dual Hormone Pump Therapy

A convenient way to deliver both insulin and C-peptide would be to utilize an already existing insulin delivery technology, the insulin pump. Insulin pumps deliver the hormone continuously through subcutaneous administration [23]. This continuous delivery allows for better insulin control than injections alone. In fact, use of an insulin pump is better at lowering A1C, controlling glycemic levels, and reducing severe hypoglycemia in patients [23,114]. Insulin pumps could also deliver C-peptide in equimolar concentrations subcutaneously, which would more closely approximate physiological hormone delivery in patients without T1DM. Still, use of the insulin pump comes with the risk of increased hypoglycemic episodes since the pump is unable to control the release of insulin based on blood glucose levels [115]. This could also lead to an excess of C-peptide being released.

### 5.2. Islet or Whole Pancreas Transplantation

Transplantation of isolated islets or the whole pancreas is an alternative treatment option to replace islet beta cells that would provide both insulin and C-peptide endogenously and may provide a more physiological treatment for patients with T1DM than exogenous insulin replacement alone. This procedure replaces the destroyed beta cells and allows for the continuous normalization of blood glucose levels. Since the patient would now have functional islets, there would be no further need of constant monitoring of blood glucose levels and insulin injections. Likewise, this allows for the production of C-peptide in addition to insulin, thus addressing the issue of dual hormone deficiency.

Transplantation of the whole pancreas is a definitive long-term treatment option for many patients with T1DM and selected patients with T2DM. The majority of pancreas transplants are performed in conjunction with a kidney transplant (approximately 80% performed simultaneously with a kidney transplant in diabetic and uremic patients, 15% after a kidney transplant in diabetic and posturemic patients), while very few (approximately 8%) are performed as pancreas transplants alone in brittle diabetic nonuremic patients [116]. Pancreas graft survival has significantly improved over the last decade. For instance, the one-year graft survival percentage for pancreas transplant alone, pancreas after kidney transplant, and simultaneous pancreas kidney transplant are 74.3%, 78.7%, and 85.8%, respectively. Simultaneously, the overall graft half-life has improved from 7.9 years in 1991 to 12.8 years in 2010 [117]. Whole pancreas transplants restore normoglycemia almost immediately after transplantation and patients were found to experience prevention and even reversal of diabetes associated complications [118,119]. Despite these promising results, whole pancreas transplantation is a major invasive surgery that may lead to surgical complications.

Islet transplantation, on the other hand, is associated with a lower morbidity rate as a compared to whole pancreas transplantation. Transplantation of human islets, using a precise chronic immunosuppressive regimen restored patients to normoglycemia with 80% remaining insulin independent after one year [120,121]. With a decade of follow-up, the Edmonton group reported that 79% of patients had full or partial graft function with correction of HbA1c and 35% were insulin independent for almost three years [122]. Collectively, there are now at least five independent human islet transplant centers reporting 50 to 80% insulin independence at four years [123]. While further study is needed, preliminary results after islet transplantation are similar to whole pancreas transplant and indicate stabilization or improvement in kidney and heart function, vascular complications, retinopathy, and neuropathy in patients that received simultaneous kidney and islet grafts [115,124].

Still, the pancreas, especially the pancreatic islets, are extremely delicate. If the organ is not handled correctly or expeditiously, the exocrine tissue of the pancreas can quickly release digestive enzymes that will break down the organ and destroy the islets. As multiple donors are needed to acquire enough islet cells for a single patient awaiting transplantation, and as the number of organ donors is severely lacking as compared to patients awaiting transplantation, pancreatic tissue is difficult to come by [125,126]. Moreover, transplantation requires continuous administration of immunosuppressive drugs, which are necessary to prevent rejection of the transplanted tissue. These drugs can cause a variety of complications such as increased infections and risk of cancer, extended wound healing, organ toxicity, and sometimes even destruction of the transplanted tissue [127].

### 5.3. Pancreatic Islet Encapsulation

Encapsulation of pancreatic islet transplants would remove the requirement, and thus the complications, of chronic immunosuppressive drugs. Encapsulation involves encasing the pancreatic islets within a semi-permeable membrane, often composed of alginate, agarose, chitosan, or other polymers [128]. This porous membrane creates a physical barrier between the recipient’s immune system and the transplanted islets. The goal of encapsulation is to sequester the islets from the immune system while still allowing for the passage of nutrients, glucose, and oxygen, into the capsule and the passage of insulin and waste out of the capsule [129]. This would address the issue of islet rejection, and toxic immune suppression, meanwhile permitting endogenous insulin production by the transplanted islets.

Many different encapsulation approaches are under study to achieve this goal including nanoencapsulation (coating the islets using biomaterials with opposing charges), microencapsulation (encapsulating small islet aggregates), and macroencapsulation (encapsulating a large cluster of islets) [129]. Additionally, many different encapsulation materials are under development to decrease fibrotic and inflammatory responses [129].

These issues are important and must be addressed since encapsulated islets receive inadequate levels of oxygen and nutrients due to inflammation and fibrosis at the capsule site and clumping of transplanted capsules which hinders blood vessel growth [130]. Without blood vessel access, oxygen, hormones, glucose, and nutrients are limited to passive diffusion across the porous membrane. This delays the ability of the transplanted islets to sense glucose levels and release the appropriate amount of insulin in a timely manner. Additionally, the capsule material itself along with proteins released from the transplant elicit an inflammatory response similar to a delayed hypersensitivity reaction [131]. Macrophages are often seen surrounding the encapsulated islets leading to elevated proinflammatory cytokine levels, which increases leukocyte infiltrate and causes further inflammation in the area. Lastly, encapsulated islets transplanted into the peritoneal cavity often clump together which broadens the inflammatory response and sequesters the innermost islets [131]. This further exasperates the problem of oxygen availability, leading to transplant cell death. Strategies to prevent host fibrotic response, inflammation, clumping, and increased blood vessel access are currently under investigation [129].

### 5.4. Sertoli Cells

A novel method to deliver insulin and C-peptide is with Sertoli cells (SCs). SCs are immune-privileged cells found in testes that function to sustain and protect male germ cells from autoimmune destruction. This is necessary as the advanced germ cells express novel antigens that if exposed to the immune system can lead to immune cell activation. Not only do SCs create a physical barrier to protect the germ cells, SCs also produce and secrete immunomodulatory factors that aid in the establishment of this immune-privileged environment [132].

Researchers have taken advantage of these immunoregulatory properties and cotransplanted pancreatic islets with SCs under the kidney capsule where it has been shown that SCs prolong islet graft survival when transplanted as allo- or xenografts, and even into non-obese diabetic (NOD) autoimmune mice [112,133,134]. For example, cotransplantation of pancreatic islets and SCs isolated from immune competent BALB/c mice into streptozotocin-treated diabetic C3H mice significantly prolonged islet allograft survival (mean graft survival time 61.7 ± 6.9 days), whereas islets transplanted alone were all rejected by the host immune system (mean graft survival time 26.9 ± 2.1 days) [135]. While all the islet-alone grafts rejected within 34 days, 58.8% of the SC/islet co-grafts survived and remained normoglycemic throughout the study with several grafts collected at over 100 days post-transplantation containing insulin-positive islets adjacent to SCs organized into tubule-like structures [135]. Furthermore, the islets were protected without the use of harsh immunosuppressive therapies.

Since SCs transplanted as allo- or xenografts survived long-term (at least 100 days), this suggested they could be used as an alternative vehicle to deliver insulin and C-peptide. Therefore, SCs were engineered to express insulin and C-peptide [136,137]. Using an adenoviral vector containing furin-modified human proinsulin cDNA, SCs from pigs, mice, and rats were transduced to express insulin and C-peptide. These SCs were then transplanted into diabetic mice and blood glucose levels were monitored. Normoglycemia was achieved within one day after transplantation and blood glucose levels remained significantly lowered for four to five days post-transplantation [136]. Use of an adenoviral vector resulted in high expression of insulin. However, stable integration of the insulin gene was not achieved and so the insulin expression was transient as indicated by loss of insulin expression by surviving SCs. Nevertheless, these results indicate that SCs have the potential to be engineered to express biologically active insulin and C-peptide at relevant levels.

To address the issue of transient insulin expression, we created a lentiviral vector that was modified to express high levels of insulin [138]. MSC-1 cells (a mouse SC cell line) were transduced with this lentiviral vector containing furin-modified mouse proinsulin cDNA and found to stably produce insulin. After transplantation as allografts in diabetic BALB/c mice, 100% of grafts collected at day 50 (7/7) and at days 70, 80, and 85 contained surviving MSC-1 cells. Additionally, 85.7% (6/7) of grafts collected at day 50 and 66.6% (2/3) of grafts collected at day 70 or higher were positive for insulin protein. Moreover, there was a significant decrease in blood glucose levels in both diabetic SCID and BALB/c mice for up to four days after transplantation with these cells. Interestingly, in three of the SCID mice and one of the BALB/c mice blood glucose levels decreased again by days 50 and 70, respectively. Overall, these studies support the hypothesis that SCs can be engineered to deliver therapeutic proteins, like insulin, to treat chronic diseases, like diabetes.

**Figure 7:**
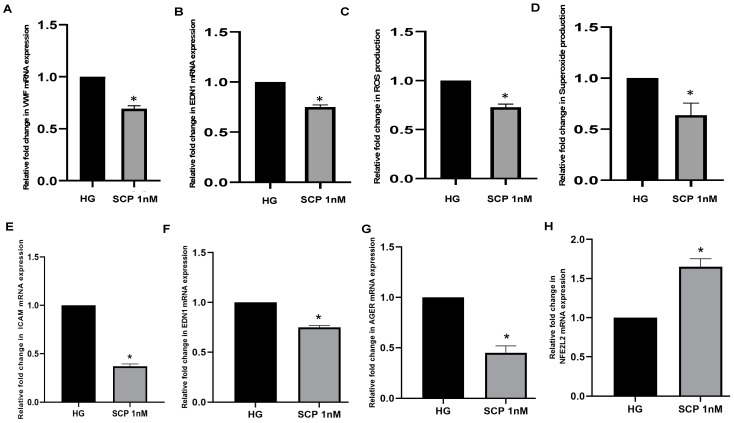
SC secreted C-peptide decreases endothelial dysfunction, activation and oxidative stress. Methods: PPECs (**A**–**D**) or HUVECs (**E**–**H**) were cultured for 4 h in high glucose (HG; 33 mmol/L _D_-glucose) or HG media containing NPSC (**A**–**D**) or MSC-1 (**E**–**H**) media with 1 nM of C-peptide (SCP). Real time PCR for VWF (**A**), EDN1 (**B**,**F**), ICAM (**E**), AGER (**G**) and NFE2L2 (**H**) was performed using mRNA isolated from treated PPECs or HUVECs. GAPDH was used as an endogenous control and the fold change for the gene of interest was calculated relative to the level in the reference sample (cells cultured in HG media). Oxidative stress was measured by quantifying levels of ROS (**C**) and superoxide (**D**). The fold change in the levels of ROS and superoxide was calculated relative to the level in the reference sample (cells cultured in HG media). Unpaired Student’s *t*-test (* *p* < 0.05) was used to determine significant differences as compared to HG.

In a preliminary attempt to test the effects of SC secreted C-peptide on endothelial cells, we cultured pig pulmonary endothelial cells (PPECs) and human umbilical vein endothelial cells (HUVECs) with media collected from SCs engineered to express insulin and C-peptide. First, we measured the amount of C-peptide secreted from genetically engineered MSC-1 cells and neonatal porcine SCs (NPSCs), and demonstrated that engineered MSC-1 cells and NPSCs secreted 165 nM and 327 nM of C-peptide, respectively. To analyze the effects of SC-secreted C-peptide on endothelial cells, PPECs or HUVECs cultured in high glucose (HG; to mimic hyperglycemic state in DM) were treated with NPSC or MSC-1 cell supernatant containing C-peptide (1 nM), respectively. PPECs or HUVECs cultured in HG alone were used as controls. Interestingly, addition of SC supernatant containing C-peptide effectively decreased hyperglycemia-induced endothelial dysfunction in both PPECs and HUVECs as compared to HG controls (Figure 7). For instance, NPSC-secreted C-peptide significantly decreased the mRNA expression of VWF and EDN1. NPSC-secreted C-peptide also significantly decreased oxidative stress, as demonstrated by decreased levels of ROS and superoxide. Similarly, MSC-1 cell-secreted C-peptide resulted in significantly decreased expression of ICAM-1, EDN1, AGER and an increased level of master antioxidant genes, NFE2L2. Collectively, while further study is needed, our data suggests that SC-secreted C-peptide along with insulin have beneficial effects in decreasing the hyperglycemia-induced endothelial dysfunction, activation, and oxidative stress.

Utilizing SCs in transplantation procedures may lead to better methods to extend transplant viability. However, we still do not fully understand the mechanisms SCs use in immunosuppression. Moreover, since this is a cell therapy, more research needs to be conducted to ensure that SC cotransplantation is safe for patients. Further research should focus on these potential concerns.

## 6. Discussion

Diabetes and diabetes-related complications account for roughly 12% of all deaths in the US [3,7]. With over 500 million people diagnosed with diabetes and prediabetes globally, this is an imperative worldwide concern [1]. Diabetes-related deaths often involve circulatory microvascular and macrovascular diseases such as CVD, stroke, and kidney failure. These complications occur far more often in patients with T1DM. One mechanism greatly contributing to these comorbidities is hyperglycemia-related endothelial dysfunction and activation, which includes excess production of ROS, chronic inflammation, recruitment of leukocytes, vasoconstriction, and thrombosis.

As patients with T1DM no longer have insulin-producing pancreatic beta cells, they no longer produce C-peptide. C-peptide has been shown to decrease these effects when delivered in conjunction with insulin as a treatment for T1DM, and offers a potential therapy for T1DM patients to decrease the risk of cardiovascular comorbidity development [44,82,85,86]. Dual hormone therapy, either with a pump containing both insulin and C-peptide or by transplantation of pancreatic islets, is one option to deliver both hormones. Another novel idea involves the use of immune privileged SCs, either by cotransplantation with islets or as a cell therapy where engineered SCs produce insulin and C-peptide. This offers potential treatment options to achieve normoglycemia without the use of harmful immune suppressive drugs, and additionally, allows for endogenous production of these proteins [139,140].

## Figures and Tables

**Table 1:** Summary of C-Peptide Therapy Studies.

Study	Model	C-Peptide Delivery	Results	Ref.
In vivo	STZ ^1^-induced diabetic rats	Injection with biosynthetic human C-peptide 2×/day for 5 weeks	Improvement of vascular and neural dysfunction by preventing sodium potassium ATPase disruption	[78]
In vitro	Rat arterioles from cremaster muscles	Biosynthetic human C-peptide (0.3–1000 ng/mL doses)	Increased arteriolar dilation through NO-mechanism	[79]
In vitro	Bovine aortic endothelial cells	Human C-peptide (0.033–66.6 nM)	Increased eNOS activity and NO synthesis through increased calcium concentrations intracellularly; increased blood flow to extremities and skin	[80]
In vitro	Bovine pulmonary aortic endothelial cells and human erythrocytes	Human C-peptide (20 nM)	C-peptide plus zinc stimulated NO production through erythrocyte mediation	[81]
Clinical Study	T1DM patients	Intravenous administration of C-peptide with insulin for one hour	Increased capillary blood flow velocity to extremities and skin	[82]
In vivo	Male Sprague Dawley rats	Intravenous administration of bolus of biosynthetic human C-peptide (7 or 70 nmol/kg)	Decreased inflammation by inhibiting endothelial-leukocyte interaction through decreased endothelial cell surface expressions of P-selectin and ICAM-1 by a NO-dependent mechanism	[83]
Clinical Study	T1DM and T2DM patients	Comparison of ATPase activity and C-peptide levels between T1DM and T2DM patient groups	Lower C-peptide levels correlated with lower erythrocyte sodium potassium ATPase activity	[84]
Ex vivo	T1DM patient and healthy control blood samples	Preincubation of erythrocytes with proinsulin C-peptide (0–66.6 ng/L)	Improvement of erythrocyte deformability	[85]
Ex vivo	T1DM patient and healthy control blood samples	Incubation of blood samples with human C-peptide or C-peptide fragments (6.6 nM)	Improvement of erythrocyte deformability	[86]
In vitro	Human umbilical vein endothelial cells	Incubation with C-peptide (0.5 nM)	Decreased ROS production through inhibition of intracellular VEGF mechanism	[87]
In vivo	STZ ^1^-induced diabetic mice	Injected with C-peptide (2 μL) into eye	Decreased vascular permeability; decreased microvascular leakage in back skin and retina	[87]
In vivo	Male C57BL/6 mice	Injected with zinc gluconate (1.3 mg/kg) daily for three days before infection, then injected with of C-peptide (280 nmol/kg)	Zinc availability before polymicrobial infection is necessary for C-peptide’s anti-inflammatory functions through management of NF-κB pathways	[88]
In vivo	STZ ^1^-induced diabetic rats	Intravenous administration of human C-peptide (0.5 nmol/kg per minute) for 140 min	Reduced glomerular hyperfiltration rate, reduced glomerular protein leakage, and restored half of normal renal functional protein reserve	[89]
In vivo	STZ ^1^-induced diabetic rats	Intravenous administration of rat C-peptide II (50 pmol/kg per minute) for 14 days	Prevented glomerular hypertrophy, reduced glomerular hyperfiltration rate, prevented albuminuria	[90]
In vivo	STZ ^1^-induced diabetic rats	Subcutaneous infusion of rat C-peptide II (50 pmol/kg per minute) for four weeks	Prevented glomerular hypertrophy, reduced mesangial matrix expansion of diabetic nephropathy	[91]
Clinical Study	T1DM patients	Initial intravenous administration of C-peptide overnight, then two infusions of C-peptide (5 and 30 pmol/kg per minute) for one hour	Reduced glomerular filtration rate, increased effective renal plasma flow, increased whole-body glucose utilization	[92]
Clinical Study	Normotensive patients having micro- albuminuria	Daily subcutaneous injection of human C-peptide (600 nmol) with regular insulin treatment for three months	Improved glycemic control, decreased urinary albumin excretion, decreased nerve dysfunction	[93]
In vivo	STZ ^1^-induced diabetic rats	Subcutaneous osmotic minipump implants with rat C-peptide II (50 pmol/kg per minute)	Increased sciatic and saphenous nerve conduction velocity; improved nerve function	[94]
Ex vivo	Retroperitoneal adipose tissue from male rats	Incubated with C-peptide, insulin, or both (6nM C-peptide, 10 nM insulin)	Reduced basal lipolysis, decreased isoproterenol-stimulated lipolysis, modulated some insulin metabolic mechanisms	[95]
In vivo	Diabetic BB/Wor rats	Subcutaneous osmopump administration of rat C-peptide II (75 nmol/kg daily)	Increased neural sodium potassium ATPase activity, decreased paranodal swelling, decreased acute and chronic nerve conduction issues	[96]
Clinical Study	T1DM patients with diabetic polyneuropathy symptoms	Intravenous administration of human C-peptide for 3 h (0.11–1.73 nmol/L)	Increase of respiratory heart rate variability; improved autonomic nerve function	[97]
Clinical Study	T1DM patients without peripheral neuropathy symptoms	Four daily doses of C-peptide (600 nmol/day)	Increased function of sensory nerve conduction velocity; improved vibration perception	[98]
Ex vivo	CD4 T cells from healthy individuals	CD4 T cells were incubated with recombinant C-peptide (10 nM for 2.5 h)	Stimulation of T cell chemotaxis involving proinflammatory pathways	[99]
Ex vivo	Thoracic artery tissue from T2DM patients	Immunohistochemical staining for C-peptide and macrophages	Accumulation of C-peptide colocalized with monocytes and macrophages in thoracic arterial blood vessel wall of T2DM patients in early atherogenesis;	[100]
In vitro	Swiss 373 mouse fibroblast cell line	Incubated with mouse C-peptide (1 nM for 24 h)	Activation of PKC/IκB/NF-κB inflammatory signaling pathways	[101]

^1^ Streptozotocin.

## Abbreviations

ACE: angiotensin-converting enzyme, ACE2: angiotensin-converting enzyme 2, AGE: advanced glycation end-product, AGER: advanced glycation end-product receptor, Ang I: angiotensin I, Ang II: angiotensin II, AT1: angiotensin II type 1, AT2: angiotensin II type 2, CAD: coronary artery disease, CAM: cell adhesion molecule, CD62E: E-selectin, CD62P: P-selectin, CVD: cardiovascular disease, DAG: diacylglycerol, DKA: diabetic ketoacidosis, DM: diabetes mellitus, EDN1: endothilin-1, eNOS: endothelial nitric oxide synthase, ERSD: end stage renal disease, FA: fatty acid, GI: gastrointestinal, GPCR: G-protein coupled receptor, GPX1: glutathione peroxidase 1, HG: high glucose, HUVEC: human umbilical vein endothelial cell, ICAM-1: intracellular adhesion molecule-1, IL-1B: interleukin-1B, IR: insulin resistance, LDL: low-density lipoprotein, LEA: lower extremity amputation, MAS: MAS1 oncogene, MSC-1: mouse Sertoli cell line-1, Mϕ: macrophage, NADPH: nicotinamide adenine dinucleotide phosphate, NFE2L2: nuclear factor erythoid 2-related factor 2, NF-κB: nuclear factor-κB, NO: nitric oxide, NOD: non-obese diabetic, NPSC: neonatal porcine Sertoli cell, PAI-1: plasminogen activator inhibitor-1, PC: prohormone convertase, PKC β: protein kinase C beta, PPEC: pig pulmonary endothelial cell, ROS: reactive oxygen species, SC: Sertoli cell, SOD1: superoxide dismutase, STZ: streptozotocin, T1DM: type 1 diabetes mellitus, T2DM: type 2 diabetes mellitus, US: United States, VCAM-1: vascular cell adhesion molecule-1, VEGF: vascular endothelial growth factor, VWF: von Willebrand factor.
